# Editorial: Autonomic dysfunction in multiple system atrophy

**DOI:** 10.3389/fneur.2022.1048895

**Published:** 2022-11-03

**Authors:** Tatsuya Yamamoto, Maria Teresa Pellecchia, Ryuji Sakakibara

**Affiliations:** ^1^Department of Rehabilitation Sciences, Chiba Prefectural University of Health Sciences, Chiba, Japan; ^2^Department of Neurology, Graduate School of Medicine, Chiba University, Chiba, Japan; ^3^Department of Medicine, Surgery and Dentistry “Scuola Medica Salernitana”, Neuroscience Section, University of Salerno, Salerno, Italy; ^4^Neurology Division, Department of Internal Medicine, Sakura Medical Center, Toho University, Sakura, Japan

**Keywords:** multiple system atrophy, REM sleep behavior disorder, cardiovascular autonomic failure, cognitive dysfunction, movement-related cortical potential

Multiple system atrophy (MSA) is clinically characterized by the combination of autonomic, cerebellar, and extrapyramidal dysfunction. The presence of autonomic dysfunction is mandatory for the diagnosis of MSA. However, autonomic dysfunction in MSA patients may not be easily recognized by general neurologists.

The new Movement Disorder Society (MDS) diagnostic criteria for MSA were published in April 2022 (1). Concerning genitourinary autonomic dysfunction, “unexplained voiding difficulties with post-void urinary residual volume” have been added to core clinical features of clinically established and probable MSA. A cutoff at >100 ml post-void urinary residual volume was set in the criteria of clinically established MSA. Regarding cardiovascular autonomic dysfunction, neurogenic orthostatic hypotension is defined as a ≥20 mmHg systolic blood pressure (SBP) drops usually accompanied by a diastolic BP (DBP) drop of ≥10 mmHg and a Δheart rate (HR)/ΔSBP ratio < 0.5 beats per minute (bpm)/mmHg within 3 min of standing or head-up tilt test (HUT) using oscillometric measurements. The classical ≥20/10 mmHg BP drop within 3 min in the upright position criterion for neurogenic orthostatic hypotension has better sensitivity for the diagnosis of MSA compared to the ≥30/15 mmHg drop.

BP drop criterion of the second consensus criteria for the diagnosis of MSA was published in 2008 with similar specificity (2). Furthermore, REM sleep behavior disorder (RBD) is highly prevalent in MSA, and polysomnography (PSG) proven RBD is an essential feature of possible prodromal MSA in the new MDS diagnostic criteria for MSA.

The goal of this Research Topic is to shed light on autonomic dysfunctions in MSA for all neurologists.

Although many autonomic tests were reported to be useful in the diagnosis of MSA, HUT and PVR measuring using ultrasonography might be the most important autonomic tests, because criteria of autonomic dysfunctions in clinically established and probable MSA depend on the severity of orthostatic hypotension and PVR (1, 3, 4). Furthermore, HUT and measuring PVR using ultrasonography might be easily performed in most institutions (3). Normal cardiac sympathetic imaging (123I-MIBG-scintigraphy) and supine plasma noradrenaline level >100 pg/ml associated with neurogenic OH might support the diagnosis of MSA (1).

Concerning lower urinary tract dysfunction, MSA patients usually show urinary urge incontinence and voiding difficulties with significant PVR, which could be represented as detrusor hyperactivity with impaired contraction in the urodynamic study (1). Detrusor sphincter dyssynergia is also found in MSA patients, which might mean the degeneration of the spinal autonomic nervous system in MSA (5). Unexplained abnormal sphincter electromyography (EMG) represents the neurodegeneration in Onuf's nucleus. Although urodynamic study and sphincter EMG are invasive examinations and are sometimes difficult to perform in some institutions, these tests can validate the lower urinary tract dysfunction in MSA.

Kermorgrant et al. examined the gender and age differences in autonomic failure in MSA using transgenic mice overexpressing human alpha-synuclein under the control of the oligodendrocyte-specific proteolipid promoter (PLP-α-syn). They reported that baroreflex sensitivity was significantly changed in PLP-α-syn mice and was age-dependent. An impaired heart rate variability (HRV) was observed at 12 months of age in PLP-α-syn female but not in male mice.

Lazzeri et al. aimed to evaluate cognitive performance and autonomic dysfunctions in MSA-P and MSA-C and to compare the results of these two groups. The author concluded that MSA-C patients reached lower scores in tests of executive and verbal memory, and no statistically significant difference in cardiovascular autonomic parameters was identified between MSA-P and MSA-C patients. They also suggested that their findings do not support the role of dysautonomia as a major driver of cognitive differences between MSA-P and MSA-C.

Yang et al. examined the Bereitschaftpotential (BP) in MSA. The BP is defined as the pre-movement session of the movement-related cortical potential (MRCP) which represents the cortical neuronal activity corresponding to intentional movement. Bilateral pre-supplementary motor area (pre-SMA) activation contributes to early BP (usually arising from 1,500 to 500 ms before movement onset) and the subsequent SMA proper, pre-motor and primary motor cortical activation contralateral to the movement contribute to late BP (usually arising 500 to 0 ms before movement onset). The authors found that the late BP amplitude was significantly reduced in the contralateral parietal area in both MSA-C and MSA-P groups. The MSA-C group exhibited a more extensive reduction of the late BP amplitude than the MSA-P group. The differences in early BP between MSA-P, MSA-C, and control groups were not robust. Because patients with Parkinson's disease (PD) had a profound early BP reduction in the frontotemporal area, their findings imply that different phases of volitional movement preparation are affected in PD and MSA.

Giannini et al. provided a narrative review paper regarding the RBD in MSA patients. RBD is one of the most robust markers of an underlying alpha-synucleinopathy and a large corpus of literature documented the high prevalence of RBD in MSA. However, few studies have systematically investigated the prevalence of RBD as a mode of disease onset and its role in disease progression. The authors concluded that almost all MSA patients experience RBD at some point in the disease course. The prevalence of RBD as the first symptom of disease onset ranged from 10 to 60%. The authors also reported that MSA patients who presented with RBD at disease onset showed a more rapid and severe disease progression, mainly due to a rapid involvement of autonomic nervous system and early achievement of milestones of disease progression.

In conclusion, this Research Topic might be helpful for readers to increase their knowledge of the clinical characteristics of autonomic involvement and the utility of autonomic function tests in diagnosing and management of MSA.

## Author contributions

TY wrote the manuscript. MP and RS supervised the manuscript. All authors contributed to the article and approved the submitted version.

## Conflict of interest

The authors declare that the research was conducted in the absence of any commercial or financial relationships that could be construed as a potential conflict of interest.

## Publisher's note

All claims expressed in this article are solely those of the authors and do not necessarily represent those of their affiliated organizations, or those of the publisher, the editors and the reviewers. Any product that may be evaluated in this article, or claim that may be made by its manufacturer, is not guaranteed or endorsed by the publisher.
